# An EIAV field isolate reveals much higher levels of subtype variability than currently reported for the equine lentivirus family

**DOI:** 10.1186/1742-4690-6-95

**Published:** 2009-10-20

**Authors:** Jodi K Craigo, Shannon Barnes, Baoshan Zhang, Sheila J Cook, Laryssa Howe, Charles J Issel, Ronald C Montelaro

**Affiliations:** 1 Center for Vaccine Research, University of Pittsburgh, Pittsburgh, PA 15261, USA; 2 Department of Microbiology and Molecular Genetics, University of Pittsburgh, Pittsburgh, PA 15261, USA; 3 Department of Veterinary Science, University of Kentucky, Lexington, Kentucky, 40516, USA

## Abstract

**Background:**

Equine infectious anemia virus (EIAV), a lentivirus that infects horses, has been utilized as an animal model for the study of HIV. Furthermore, the disease associated with the equine lentivirus poses a significant challenge to veterinary medicine around the world. As with all lentiviruses, EIAV has been shown to have a high propensity for genomic sequence and antigenic variation, especially in its envelope (Env) proteins. Recent studies have demonstrated Env variation to be a major determinant of vaccine efficacy, emphasizing the importance of defining natural variation among field isolates of EIAV. To date, however, published EIAV sequences have been reported only for cell-adapted strains of virus, predominantly derived from a single primary virus isolate, EIAV_Wyoming _(EIAV_WY_).

**Results:**

We present here the first characterization of the Env protein of a natural primary isolate from Pennsylvania (EIAV_PA_) since the widely utilized and referenced EIAV_WY _strain. The data demonstrated that the level of EIAV_PA _Env amino acid sequence variation, approximately 40% as compared to EIAV_WY_, is much greater than current perceptions or published reports of natural EIAV variation between field isolates. This variation did not appear to give rise to changes in the predicted secondary structure of the proteins. While the EIAV_PA _Env was serologically cross reactive with the Env proteins of the cell-adapted reference strain, EIAV_PV _(derivative of EIAV_WY_), the two variant Envs were shown to lack any cross neutralization by immune serum from horses infected with the respective virus strains.

**Conclusion:**

Taking into account the significance of serum neutralization to universal vaccine efficacy, these findings are crucial considerations towards successful EIAV vaccine development and the potential inclusion of field isolate Envs in vaccine candidates.

## Background

Equine Infectious Anemia Virus (EIAV), a macrophage-tropic lentivirus of the family Retroviridae, causes a persistent and potentially fatal infection in equids and a chronic disseminated disease that is of worldwide importance in veterinary medicine (reviewed in Craigo, et al. 2008 and Leroux et al. 2004). Natural and experimental infection with EIAV results in a rapid and dynamic disease process that differs markedly from the slowly progressive degenerative diseases associated with other lentiviral infections including HIV-1 infection of humans. EIAV infection can be transmitted via iatrogenic sources such as contaminated syringe needles, but is predominantly spread by blood-feeding insect vectors (mainly horseflies and deerflies). Hence, disease is most problematic in regions with warmer climates [1,2]. The actual number of infected animals in various geographical regions is not precisely known due to a lack of routine testing. Since its inception, testing in the United States has generally increased on an annual basis [3], but the number of animals tested still represents a small proportion of the total equine population.

EIA disease in equids emerges as a vigorous progression through three stages: acute, chronic, and inapparent. The acute and chronic stages of EIA are defined by episodes of clinical disease that are triggered by waves of viremia and distinguished by fever, anemia, thrombocytopenia, edema, diarrhea, lethargy, and various wasting signs. By 8-12 months post-infection, horses typically progress to life-long (long-term) inapparent carriers, presumably due to the development of enduring protective host immunity [4]. These inapparent carriers, however, remain infected for life with the maintenance of markedly different levels of steady state virus replication in monocyte-rich tissue reservoirs [5-7]. Stress or immune suppression of EIAV inapparent carriers can induce an increase in viral replication and potentially a recrudescence of disease [7-9]. Thus, EIAV offers a unique model for characterizing natural immunologic control of lentivirus replication and disease, for elucidating the nature and role of viral variation in persistence and pathogenesis, and ultimately for developing and modeling lentiviral vaccines.

Among virulent lentiviruses, EIAV is unique in that greater than 90% of infected horses progress from a chronic disease state to an inapparent carrier stage despite aggressive virus replication and associated rapid antigenic variation. However, the United States Department of Agriculture (USDA) along with state animal regulatory agencies require euthanasia or strict lifelong quarantine for EIAV seropositive horses. Within the US, each state drafts its own requirements with reference to EIAV and the movement of horses as well as changes in ownership of horses. All seropositive horses must be registered with the state veterinarians and the federal Animal and Plant Health Inspection Service (APHIS) office [3,10]. Given EIAV's role as an animal model for HIV vaccine studies, the associated costs of equine testing, and the general issue of equine health, the development of an effective EIAV vaccine holds a multifaceted significance. Like all lentiviruses, the roadblock to effective vaccine development for EIAV is the high level of antigenic variation that occurs during viral replication throughout all stages of infection and disease.

Studies of EIAV variation during persistent infection in experimentally infected equids have clearly identified characteristic changes in envelope sequences that alter viral antigenic properties, evidently as a result of immune selection [11-14]. The predominant site of EIAV variation during persistent infection is the gp90 surface envelope glycoprotein. The pattern of gp90 nucleotide and amino acid variation has been analyzed to define distinct conserved and variable protein domains [13,15-17] as observed with other animal and human lentiviruses [13,18-21]. Variation of the EIAV envelope gene has therefore served as a distinct marker for analysis of viral population evolution and can hence be utilized as a marker of variant isolates.

Despite the worldwide prevalence of EIAV infections, experimental studies to date have centered on relatively few viral isolates. Analyses of viral pathogenesis have essentially focused on a strain of EIAV termed Wyoming (isolated in North America) and its derivatives while a minority of reported studies have utilized a Chinese variant. In fact, in the last thirty years, 97% of published studies on EIAV natural isolates have been based on the Wyoming isolate directly, or on *in vivo/in vitro *derivatives of this strain (based on a PubMed search on EIAV natural isolates or experimental derivatives of those isolates within the last 30 years: approximately 548 publications; the percentage of the overall number published for each "strain" was calculated). The variant nature of the antigenicity of EIAV which has thus far obstructed successful vaccine development mandates that a larger pool of viral strains be analyzed both for consideration of pathogenesis and determination of immune correlates of protection.

In the current study, we report on the characterization of the Env genomic sequences of an EIAV field isolate recovered from a long-term inapparent carrier in the state of Pennsylvania in the United States. The observed variation of the EIAV_PA _Env compared to published EIAV isolates indicates that the current understanding of genomic divergence is greatly underestimated. Further, functional analyses of how the gp90 variation affected antigenic specificity demonstrated that the observed genomic alterations rendered the isolate neutralization distinct to immune sera from horses experimentally infected with a Wyoming-derived virus strain (EIAV_PV _[22-24]). The observations of extensive Env variation and neutralization differences in a primary EIAV isolate indicate the need for EIAV vaccine strategies that can elicit enduring broadly reactive host immune responses to protect against diverse strains of virus.

## Results

### Recovery of a primary EIAV field isolate

To characterize EIAV viral populations of naturally infected horses, we contacted the local USDA office for information on regionally identified EIAV positive carriers that were under quarantine. They identified a 25-year-old Coggins positive horse that had been infected for 15 years, but had been clinically inapparent for several years. Analysis of the serum from this donor horse indicated an antibody titer of 10^4 ^in ELISA assays against the EIAV_PV _reference strain, consistent with the seropositive results also observed in AGID diagnostic assays (data not shown). Quantitative RT-PCR analysis of plasma from the donor horse revealed a viral load in the periphery of approximately 5 × 10^3 ^copies of RNA/ml plasma. To characterize the viral population of the field isolate, EIAV_PA_, viral RNA was pelleted from the plasma. We designed consensus primers (see additional file 1: Table S1) to reverse transcribe and PCR amplify the EIAV_PA _genome based on currently available sequences in the Genbank repository. RT-PCR amplification of viral RNA failed to yield products for cloning and sequencing. It has previously been demonstrated that a blood transfer performed between an inapparent carrier and an EIAV naïve equine results in febrile EIA disease [7]. We also previously demonstrated that a majority of the viral quasispecies found in the first febrile episode reflect the same genomic sequence as the infectious inoculum [11-13]. Taken together, we chose to characterize the EIAV_PA _population of the inapparent carrier via a plasma transfer between the naturally infected animal and an EIAV naïve recipient horse.

#### Clinical and virological profile of plasma transfer recipient #9807

**Figure 1:**
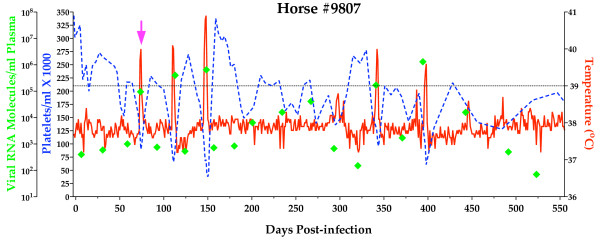
**Clinical and virological profiles of experimentally infected horse #9807**. Horse #9807 was experimentally infected with EIAV_PA _by intravenous inoculation with 5 ml of plasma from the reference Pennsylvania field isolate inapparent carrier. Rectal temperatures (red line, right Y axis) and platelet counts (blue dashed line, 1^st ^left Y axis) were followed daily for approximately 550 days (X-axis). Quantitation of the virus load (green diamond, 2^nd ^left Y axis) was performed on viral RNA extracted from plasma at periodic time points during throughout the initial infection, fever episodes and asymptomatic stages. Febrile episodes were defined by a rectal temperature above 39°C in conjunction with a reduction in the number of platelets below 100,000/μl of whole blood and other clinical signs of EIA disease. The acute phase of disease (74DPI) from which viral populations were sampled is indicated (pink arrow).

An outbred, mixed-breed naïve horse (#9807) was infected with EIAV_PA _by transfer of infectious plasma (5 ml) intravenously. The recipient horse was monitored daily for clinical signs of EIA (fever, lethargy, petechiation, diarrhea) and blood samples were taken at regular intervals for measurement of platelets, plasma virus, and EIAV-specific serum antibodies. At approximately 70 DPI the horse experienced an acute clinical EIA episode characterized by concurrent thrombocytopenia and fever accompanied by a viremic episode of 10^5 ^copies RNA/ml (Fig. 1). Over the course of the observation period (approximately 1.5 years), clinical disease progressed from acute to chronic to inapparent. Over the 550 day observation period there was a total of five fever episodes. The viral loads exhibited typical fluctuations averaging around 10^3 ^copies RNA/ml plasma in periods of steady state replication and increasing to about 10^5 ^-10^6 ^copies RNA/ml plasma during febrile episodes (Fig. 1).

#### Isolation, cloning, and sequencing of EIAV_PA _clones

To characterize the viral quasispecies of EIAV_PA_, we isolated viral RNA from plasma taken during the acute episode, or 74 days post infection (DPI), in the recipient horse. The majority of EIAV genomic variation occurs in the 3' half of the viral RNA that encodes the *envelope, rev*, and the long terminal repeat (LTR) [2,4,7]. Thus, utilizing the consensus primers described in 3.1, we RT-PCR amplified the entire 3' half (~3 Kb) of the genome. The purified fragments were cloned, and a total of 18 positive clones subjected to sequence analysis.

### Population analyses of EIAV_PA _quasispecies

**Figure 2:**
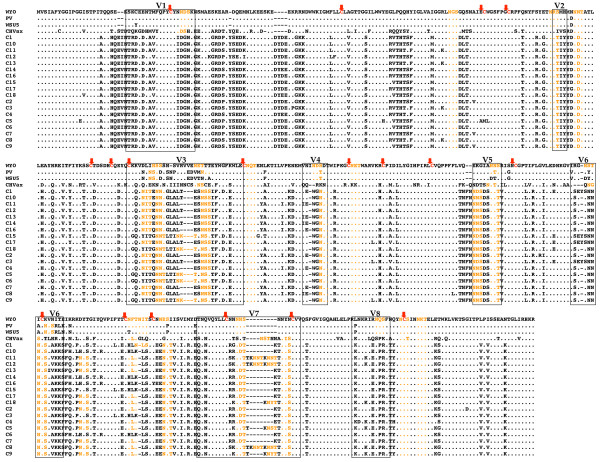
**Genomic sequences of EIAV_PA _Env gp90 quasispecies at 74 DPI**. The deduced amino acid sequences of the EIAV_PA _population and reference EIAV sequences were aligned in ClustalW to the EIAV Wyoming strain. Residues that are different from Wyoming are indicated by their single amino acid designations. Reported variable regions for the gp90 sequence are boxed. Residues identical to Wyoming sequence are indicated with (black circle). Glycosylation sites are colored orange. WYO, Wyoming; PV, EIAV_PV_; CHVax, Chinese vaccine stain; black line, absent residue; black arrow, Cysteine residues.

**Figure 3:**
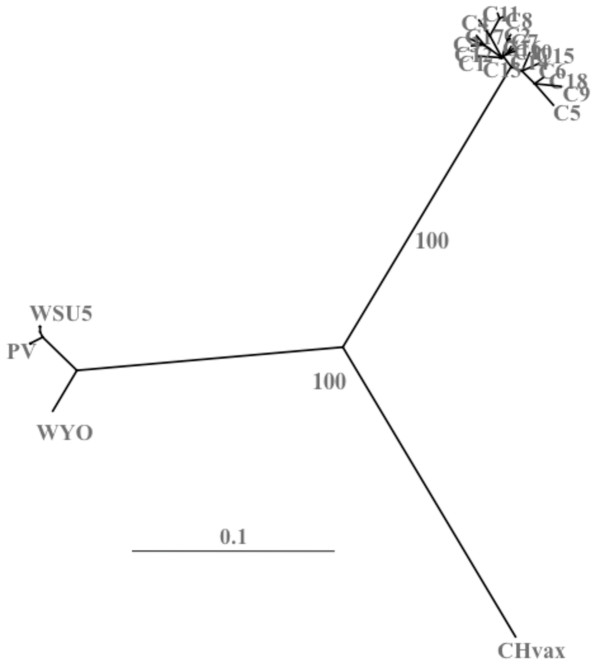
**Population characterization of horse #9807 viral envelope gp90 sequences**. A phylogenetic tree of aligned deduced amino acid sequences was constructed by the neighbor joining method from Kimura corrected distances with the optimality criterion set to distance. The tree was unrooted. Bootstrap values were determined over 1000 iterations and are indicated at the nodes of the branches. Branch lengths are proportional to the distance existing between the sequences. C"#", EIAV_PA _clone number; WYO, Wyoming; PV, EIAV_PV_; CHVax, Chinese vaccine stain.

Our primary goal was to explore natural EIAV diversity that directly affects vaccine development by examining the *env *gene, specifically the gp90 region. Three other prime regions of relevance, but not of primary significance for vaccine development, namely the *env *gp45, *S2*, and *rev *genes, as well as the LTR were also sequenced; and the results are included in the additional files 2, 3, 4 and 5. Once nucleotide sequencing was completed, the deduced amino acid sequences were visually inspected to determine the phenotype of the viral quasispecies (Fig. 2 and additional files 2, 3, 4, 5, Figs. S1-S4). Immediately, the primary observation is the vast difference in the EIAV_PA _sequences as compared to the widely utilized Wyoming-derivative EIAV_PV _and the published Chinese vaccine strain (Fig. 3). The EIAV_PA _Env sequences varied well outside of the currently designated gp90 "variable" regions [13,15,17]. Phylogenetic analyses demonstrated that the observed sequence differences between the EIAV_PA _isolates and other known EIAV strains cluster the reported Env populations and the EIAV_PA _population into a star phylogeny reminiscent of the clades distinguished in HIV-1 subtypes (Fig. 3). Calculated diversity between a consensus EIAV_PA _amino acid sequence and Wyoming gp90 was approximately 40%, compared to the current 13% maximum reported divergence among published EIAV gp90 sequences from Wyoming- derived and Chinese strains. Variations within the gp90 amino acid residues included the shifting of potential N-linked glycosylation sites among the EIAV_PA _quasispecies as compared to the Wyoming-derivative gp90 sequences. Lentiviruses utilize dense glycosylation to shield the envelope proteins from immune recognition. The number of potential glycosylation sites observed in the EIAV_PA _gp90 ranged from 15-21, depending on the individual Env clone. On average there were 19 potential glycosylation sites in the EIAV_PA _gp90, approximately 10% higher than what is observed with the Wyoming-derivative EIAV species. Notable, however, is the relative conservation of the approximate location of these glycosylation sites among the variant gp90 quasispecies. For example, in the "V3" region the EIAV_PA _population maintained three potential N-inked glycosylation sites in all variants, however, the exact location of the sites "shifted" within the respective V3 domains of the variant Env species. As observed previously, there appears to be a complete preservation of all cysteine locations in the EIAV_PA _gp90 compared to published Env sequences despite the marked variation among these gp90 species. This conservation of cysteine residues appears to be indicative of secondary structural conservation, presumably related to their role in disulfide bridges and loop formations within the gp90 protein. Furthermore, comparison of the predicted amino acid sequences determined for the gp45, Rev, and S2 proteins also reveals a conservation of critical structural features despite the substantial variation in protein sequences and high levels of average divergence (gp45, 44%, Rev, 39%, S2, 54%) from the Wyoming strain (additional files 3, 4, 5, Figs. S2-S4). For example, in gp45 all extracellular potential N-linked glycosylation sites are maintained between species although the amino acid make-up of the site may vary. Similarly, in spite of the significant differences in the amino acid sequence of EIAV_PA _Rev compared to published Rev sequences, the published RNA binding domain and nuclear export signals of Rev are conserved in EIAV_PA_.

### Characterization of EIAV_PA _envelope antigenic properties

**Figure 4:**
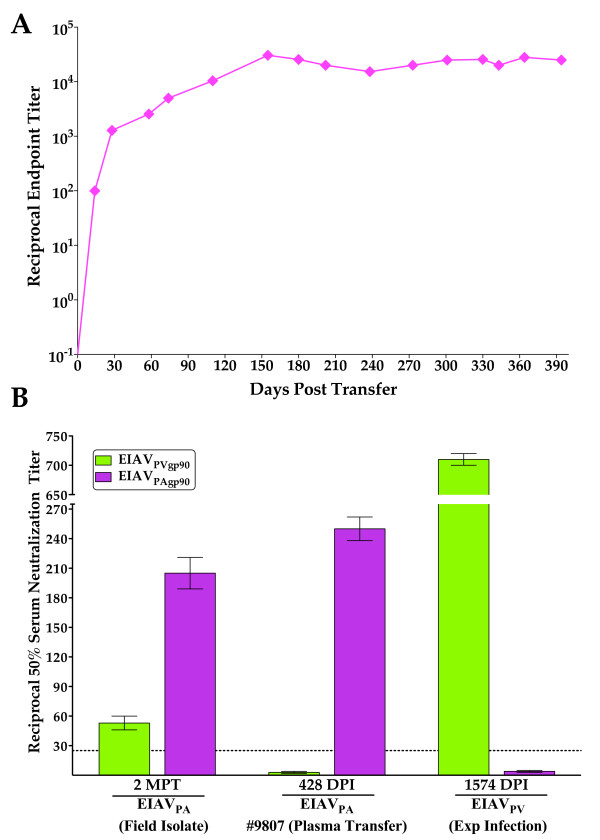
**Characterization of the Env reactivity of serum antibodies elicited by EIAV_PA _infection of horse 9807**. Envelope-specific reactivities were analyzed in both an (**A**) end-point titer assay and a (**B**) neutralization assay. (**A**) Longitudinal characterization of the quantitative properties of induced EIAV envelope-specific antibodies were conducted in ConA ELISA assay utilizing EIAV_PV _as the antigen. Mean serum antibody titers for each time point are presented as the log_10 _of the highest reciprocal dilution yielding reactivity two standard deviations above background. (**B**) The mean reciprocal dilutions of serum from infected horses that neutralized 50% of input EIAV_PV _or EIAV_PA_gp90 as measured in an infectious center assay. Serum samples included: EIAV_PA_- serum from the original Pennsylvanian field isolate plasma donor and serum from the recipient pony #9807, EIAV_PV_- serum from an experimentally infected long-term inapparent carrier. The line (dashed black line) denotes the cut off (≥ 25) value for valid 50% neutralization titers. MPT, months post transfer; DPI, days post infection.

To characterize the effects of the observed EIAV_PA _gp90 variation on antigenic properties of the Env protein, we next evaluated the Env-specific serum antibody responses of horse #9807 utilizing two separate methods, end point titer analyses (heterologous) and neutralization (homologous and heterologous) assays. We have previously characterized a complex and lengthy maturation of immune responses to viral envelope proteins during the first six to eight months post-infection that appears to be a distinctive feature of lentiviral infections as steady state infection and host immunity levels are established [25-28]. The serum of the inapparent carrier cross-reacted in ELISA with the Env proteins of our reference strain EIAV_PV _as demonstrated in earlier analyses (c.f. section 3.1). Hence, we initially characterized the development of serum antibodies in horse #9807 by longitudinal analyses of serum end-point titers to the Env protein of the reference strain EIAV_PV_. The evolution of the end-point titer of EIAV-specific serum antibodies demonstrated a characteristic development of a mature response that gradually increased throughout the first 6 months of infection, at which time the end point titer reach a steady state of approximately 10^4 ^(Fig. 4A).

We have reported a moderately slow development of serum neutralizing antibodies over a several month period following experimental EIAV infection of horses, with average maximum neutralization titers averaging 1:300 [5,27]. To examine the ability of the EIAV_PA _strain to elicit homologous and heterologous serum neutralizing antibodies, we assayed the ability of serum samples taken from horse #9807 (428 DPI) and the original Pennsylvanian inapparent carrier (two months post transfer to #9807) to neutralize EIAV_PA _and EIAV_PV _gp90 species, as presented on otherwise common proviral constructs (Fig. 4B). The neutralization activity of a reference immune serum taken from a horse experimentally infected with EIAV_PV _(1,574 DPI) was assayed in parallel as a control. Interestingly, immune sera from the experimentally EIAV_PA _and the EIAV_PV _infected horses were able to neutralize only virus containing the homologous virus gp90; there was no detectable neutralization of the virus containing the heterologous gp90 species. In contrast, however, the immune serum from the naturally infected inapparent carrier displayed neutralization activity against both the EIAV_PA _(1:200 titer) and EIAV_PV _(1:55 titer) gp90 Env species. Two-way ANOVA analyses of the neutralization results indicate a significant difference between the ability of the inapparent carrier serum to neutralize the two different gp90 Env proteins (*P *< 0.0001).

## Discussion

EIAV in addition to being an animal model for HIV/AIDS studies is a potentially fatal and economically significant infectious disease of equines found in populations of horses worldwide. We have thoroughly explored the evolution of Wyoming-derivative EIAV strains [11-14,29-35] and investigated in detail EIAV interactions with the immune system [5,28,36] as well as mechanisms of protection towards the development of a vaccine [28,36-43]. Vaccine development is essential to the global control of EIA. A common problem to all lentiviral vaccine development is the obstacle of viral evolution and more specifically viral Envelope variation and diversity.

To address this problem of Env variation and vaccine efficacy, it is essential to develop a more detailed characterization of the natural level of variation in the primary protein conferring vaccine protection, gp90. The overall level of envelope divergence observed for other common lentiviruses such as the small ruminant lentiviruses (SRLV), FIV, SIV and HIV have averaged between 10-35% [44-51]. Present understanding of the variation of EIAV has been based on a very limited number of natural field isolates. The current study of the EIAV_PA _isolate represents the first characterization of the Env protein of a natural primary isolate. The data reveal a much greater extent of Env variation than previously deduced from published Env sequences from a limited number of reference viral strains, all cell-adapted. The observed variation of the EIAV_PA _inapparent carrier population was very similar to what we have recognized in inapparent carriers from experimental infections. The level of diversity was at the same average level (data not shown) and the included phenotypic changes of a similar nature to previously observed evolution in experimental infections. While the largest amount of variation previously reported among published Env sequences indicated a maximum divergence of up to 13% variation [11,12], the EIAV_PA _gp90 sequence reveals a divergence of about 40% from EIAV_PV _and other published Env sequences. In addition to the presumed effects of this extent of Env variation on vaccine development, it is important to note that the EIAV_PA _gp90 sequence is derived from a primary virus isolate that has never been passaged in cell culture. Thus it may be assumed that the EIAV_PA _Env species may in fact be more representative of natural Env populations than the currently published Env species that are derived from virus isolated by cell culture. In this regard, the EIAV_PA _may be considered a better candidate for vaccine development compared to other cell adapted strains of EIAV.

Recently we published a report detailing the specific affects of envelope sequence variation on vaccine protection [42]. In that study we identified for the first time a significant, inverse, linear correlation between vaccine efficacy and increasing divergence of the challenge virus gp90 compared to the vaccine virus gp90 protein. The vaccine study demonstrated approximately 100% protection of immunized horses from disease after challenge by virus with a homologous gp90, but only 50% protection against challenge by virus with an Env that was 13% divergent from the vaccine strain. The calculated linear relationship predicted a complete lack of protection of immunized horses from disease upon challenge with a virus gp90 that is 23% divergent from the vaccine strain. Thus, these data suggest that the 40% divergence observed with the EIAV_PA _strain would present a substantial obstacle to the development of a broadly protective vaccine against EIA.

The extensive divergence observed between the EIAV_PA _and EIAV_PV _Env would predict differences in immunogenicity and antigenicity, including neutralization sensitivity. The current data indeed indicated distinct neutralization specificities for the two variant Env species. However, the current immune assays also indicated a substantial amount of cross reactive serum antibody as measured by ELISA assays, indicating common antibody epitopes in the variant Env proteins, despite the 40% divergence in gp90 amino acid sequences (Fig. 4A). Why this antibody cross-reactivity did not confer neutralization of variant infectious viruses remains to be determined by more rigorous characterization of the neutralization epitopes of the Env protein and the effects of sequence variation on antibody binding to and inactivation of virions.

It is of interest to note that only the immune serum from the long-term inapparent carrier displayed significant neutralization activity against both the EIAV_PA _and EIAV_PV _Env species (Fig. 4B). Since this immune serum reflects at least 25 years of persistent EIAV infection, it is possible that the broad neutralization activity is due to a maturation of antibody responses to constantly changing EIAV populations with variant Env quasispecies over this time period. While the mechanism of this cross neutralization is uncertain, it may be attributed to the collection of antibody responses to immuno-dominant type specific variable domains of the viral gp90 protein or alternatively to the slowly progressive development of antibody to immuno-recessive conserved domains of gp90. Experimental differentiation between these alternative mechanisms will provide important fundamental information relevant to the design of optimal Env protein for vaccine development.

## Conclusion

The ability of the immune serum from the long-term inapparent carrier to ultimately neutralize viruses expressing either the EIAV_PV _and EIAV_PA _gp90 protein species indicates that it is possible for the horse immune system to develop broadly neutralizing serum antibodies. Based on this observation, it will now be possible to experimentally identify the specific Env sequences that are reactive with cross-neutralizing antibodies and that may be used in vaccines to develop enduring broadly reactive antibody responses. Whether we can incorporate this new EIAV_PA _sequence information into an immunogen that can confer the level of protection observed in long-term infected animals such as the Pennsylvania animal is the challenge to vaccine development and remains to be seen. What is definite is that additional field isolates need to be evaluated in order to develop EIAV vaccines that have a chance of being broadly protective to EIAV infection.

## Methods

### Identification of a natural EIAV inapparent carrier

The USDA local office (Allegheny County, Pennsylvania) identified a naturally infected, clinically inapparent, EIAV-positive horse. The horse had been EIAV seropositive for approximately 15 years at the time of sampling, as determined by repeated serum testing in the present USDA reference AGID diagnostic assay [52]. Per Pennsylvania statutes and regulations, the horse was maintained in isolation and was under the surveillance of the local USDA Office with annual EIAV retesting. Under the supervision of the local USDA veterinarian, 500 ml of whole blood was drawn from this inapparent carrier by venipuncture into an ACD vacuum bottle. Peripheral blood mononuclear cells (PBMCs), plasma, and sera were collected and stored as previously described [13].

### Attaining an EIAV field isolate

Initial attempts to amplify and clone EIAV from the plasma of the inapparent carrier yielded insufficient levels of viral genomic PCR products (data not shown), probably due to a very low level of viremia at the time of isolation [12]. We have previously demonstrated that the viral population associated with the initial febrile episode in an experimentally infected horse fundamentally represents the species present in the infectious inoculum [11-13] and provides a much higher viral level to obtain samples for analysis. Thus, we transferred plasma from the inapparent carrier to a naïve recipient horse to amplify the viral quasispecies for subsequent isolation and characterization.

#### Experimental infection of naïve horses

An outbred, mixed-breed naïve horse (#9807) was infected with the Pennsylvanian EIAV (EIAV_PA_) field isolate by transferring 5 ml of infectious plasma from the identified naturally infected donor animal. The animal was monitored daily and maintained as described previously [13,14]. Platelet numbers were determined using the Unopette microcollection system (Becton Dickinson, Rutherford, N.J.). Clinical EIA (fever) episodes were determined on the basis of rectal temperature and platelet count in combination (rectal temperature > 39°C; platelet number < 100,000/μl of whole blood) with the presence of infectious plasma virus [2,7,13,14]. Samples of whole blood, serum, and plasma were collected weekly and daily during fever episodes. Plasma samples were stored at -80°C until used to determine plasma viral RNA level and to perform genetic analysis of viral RNA. Serum samples were stored at -20°C until being tested for antibody reactivity.

#### Field isolate viral RNA purification and amplification

Viral RNA was extracted as described previously [12,13,41]from plasma taken during the acute disease episode in the recipient horse at 74 DPI. Reverse transcription of 2 to 5 μl of purified viral RNA was performed with the SuperScriptII PreAmplification System (GibcoBRL, Rockville, MD) as previously described [13]. Multiple nested amplifications of the 3' half of the genome were performed as reported [13] using the Elongase mix (Gibco BRL, Rockville, MD). Primers for primary and nested amplifications are detailed in additional file 1, Table S1. PCR products were visualized on a 1% agarose gel prior to purification and cloning.

### Quantitative Viral RNA determinations

Plasma samples were analyzed for the levels of viral RNA per milliliter of plasma using a previously described quantitative real-time multiplex RT-PCR assay based on *gag*-specific amplification primers [53]. The standard RNA curve was linear in the range of 10^1 ^molecules as a lower limit and 10^8 ^molecules as an upper limit.

### Cloning and Sequencing

Several independent RT-PCR products (3 independent RT reactions and 8 independent nested PCR reactions) were generated (refer to 2.2.2), gel-purified using Qiagen's Qiaex (Valencia, CA), and cloned individually into the pCR2.1-TOPO^® ^vector (Invitrogen, Carlsbad, CA). Individual clones were screened by PCR. Positive colonies were consequently grown, plasmid DNA was extracted, and clones automatically sequenced (Applied Biosystems, Foster City, CA) using internal EIAV primers (see additional file 1, Table S1). DNA sequences were resolved with an ABI Prism 373 DNA sequencer (Applied Biosystems, Foster City, CA).

### Sequence Analysis

Sequences were assembled and error checked using GeneJockey II (Biosoft, Cambridge, UK). Nucleotide and deduced amino acid sequences from each clone were aligned using the ClustalW multiple sequence alignment program from the GCG Wisconsin software package and edited manually when necessary. Alignments were performed for each genomic region with the reference strains: Wyoming, Wyoming derivative strains EIAV_PV _and EIAV_WSU5_, and the Chinese vaccine strain. The amino acid divergence calculations were determined using the Kimura distance correction.

Distance analyses were conducted using Distance software as implemented in the GCG Wisconsin software package [54]. Phylogenetic analyses of sequences were constructed by the neighbor-joining method with the optimality criterion set to distance as measured in PAUP [55]. Statistical significance of branchings and clustering were assessed by bootstrap re-sampling of 1000 pseudoreplicates on the complete data set represented in a 75% majority-rule consensus tree. The tree was edited for presentation using Treeview68 K version 1.5.

### Nucleotide Sequences

All sequences have been submitted to GenBank. Nucleotide accession numbers are GBQ855742-GBQ855758.

### Construction and production of EIAV_PAgp90_

To compare the neutralization properties of the EIAV_PA _envelope to those of the EIAV_PV_, we generated a chimeric clone in which the gp90 of the representative predominant EIAV_PA _clone (associated with the first disease cycle) was substituted into the proviral backbone of our reference EIAV_UK3 _molecular clone [56] utilizing standard molecular biology techniques [57]. Briefly, the gp90 gene of clone 2 from the PA field isolates derived from horse #9807 was digested with PasI and BtsI. The purified digestion product was ligated into the EIAV_UK3 _backbone, which had also been digested with BtsI and Pas I (partial digestion with PasI). Clones were screened by sequencing using internal EIAV primers. Chimeric virus (EIAV_PA_gp90) was produced by transfecting a 4-μg sample of purified DNA from the chimeric proviral clone into 10^5 ^fetal equine kidney (FEK) cells as specified by the manufacturer of the GenePorter Transfection kit (GTS, San Diego, Calif.). The number of infectious units per ml of supernatants from transfected FEK cell cultures was then determined in a standardized infectious-center assay that uses a cell-based enzyme-linked immunosorbent assay detection system to study FEK cells [58].

### Serological Analyses

Detection of serum antibody reactivity to the EIAV capsid protein p26 was conducted using the ViraCHEK^®^/EIA kit per the manufacturer's instructions (Synbiotics Laboratory, Via Frontera, San Diego, CA). Serum samples were also evaluated for seroreactivity by the standard Coggins AGID diagnostic assay for EIA. Serum IgG antibody reactivity to EIAV envelope glycoproteins was assayed quantitatively (end point titer) using our standard concanavalin A (ConA) ELISA procedures [27]. Virus neutralizing activity to EIAV_PV _[22-24] and EIAV_PA_gp90 (refer to 2.6) mediated by immune sera was assessed in an indirect cell-ELISA based infectious center assay using a constant amount of infectious virus and sequential 2-fold dilutions of serum [27,58]. Statistical significance was calculated using GraphPad software (GraphPad software Inc., LaJolla, CA.).

## Competing interests

The authors declare that they have no competing interests.

## Authors' contributions

JKC participated in the design and directing of the study; isolated, cloned and analyzed the sequence of the viral strains; performed immunoassays and drafted the manuscript. SB performed the viral load analyses, serology and immunoassays. BZ constructed the chimeric virus clones for the immunoassays. SJC performed all procedures on the animals as well as the daily observations on the subjects. LH performed DNA isolations and provided assistance with sequencing and blood collection from field animal. CJI directed the animal studies. RCM conceived and participated in the design of the study and helped to draft the manuscript. All authors read and approved the final manuscript.

## Supplementary Material

Additional File 1 **Table S1**. This file is a table depicting the primers used to amplify and sequence the primary isolate. Click here for file

Additional File 2 **Figure S1. Genomic sequence of EIAV_PA _LTR population**. The nucleotide sequences of the EIAV_PA _population and reference EIAV sequences were aligned in ClustalW to the EIAV Wyoming strain. Residues that are different from Wyoming are indicated. Transcription factor recognition sequences are boxed. Bases identical to Wyoming sequence are indicated with (white square). WYO, Wyoming; PV, EIAV_PV_; CHVax, Chinese vaccine stain; white square, absent base. Click here for file

Additional File 3 **Figure S2. Genomic sequence of EIAV_PA _S2 population**. The deduced amino acid sequences of the EIAV_PA _population and reference EIAV sequences were aligned in ClustalW to the EIAV Wyoming strain. Residues that are different from Wyoming are indicated by their single amino acid designations. Reported predicted nucleoporin motif, SH3 binding motif, and nuclear localization signal are underlined in the Wyoming strain. Residues identical to Wyoming sequence are indicated with (white square). Predicted N-myristilation signal is in red text and boxed. Predicted CK2 phosphorylation site is in pink text and boxed. PKC phosphorylation sites are in blue text and boxes. Predicted β-sheet is indicated with (white arrow) and boxed in orange. Predicted alpha helix is indicated with (white cylinder) and boxed in yellow. All structural predictions were performed using PredictProtein . WYO, Wyoming; PV, EIAV_PV_; CHVax, Chinese vaccine stain; white square, absent residue. Click here for file

Additional File 4 **Figure S3. Genomic sequence of EIAV_PA _Rev second exon population**. The deduced amino acid sequences of the EIAV_PA _population and reference EIAV sequences were aligned in ClustalW to the EIAV Wyoming strain. Residues that are different from Wyoming are indicated by their single amino acid designations. Reported activation domain, RNA binding site and nuclear exportation signal are underlined in the Wyoming sequence and are boxed in the EIAV_PA _population and reference EIAV sequences. Residues identical to Wyoming sequence are indicated with (white square). Glycosylation sites are colored orange. WYO, Wyoming; PV, EIAV_PV_; CHVax, Chinese vaccine stain; white square, absent residue. Click here for file

Additional File 5 **Figure S4. Genomic sequence of EIAV_PA _Env gp45 population**. The deduced amino acid sequences of the EIAV_PA _population and reference EIAV sequences were aligned in ClustalW to the EIAV Wyoming strain. Residues that are different from Wyoming are indicated by their single amino acid designations. The transmembrane domain is boxed. Residues identical to Wyoming sequence are indicated with (white square). Glycosylation sites are colored orange. WYO, Wyoming; PV, EIAV_PV_; CHVax, Chinese vaccine stain; white square, absent residue. Click here for file
