# Supplementary material for: An EIAV field isolate reveals much higher levels of subtype variability than currently reported for the equine lentivirus family
Source: Retrovirology. 2009 Oct 20;6:95. doi: 10.1186/1742-4690-6-95 (PMC2770520; doi:10.1186/1742-4690-6-95)
Supplement: Additional File 1 — Table S1. This file is a table depicting the primers used to amplify and sequence the primary isolate. [file 1742-4690-6-95-S1.PDF]

Name	*Primer Sequence*	*ORF Binding Region (Polarity)*	*Method*
LS.1	GAAGAARAAYAARCAAAGACWGAAGG	Tat (sense)	PCR/Primary
LS.2	GTTATAAGGTTTGRTRYATGGG	Tat (sense)	PCR/Nested
LAS	YSAGYAGAGAATTATATTTATTACMAAGG	R region (antisense)	PCR/Both
PA1 F1	GGACTGGTGGAAAATAGGTATG	Env (sense)	Sequencing
PA1 F2	CTCTTATACGGAATTCATCC	Env (sense)	Sequencing
PA1 F3	GAGAATCCGAGATTCACATAC	Env (sense)	Sequencing
PA1 F4	GGGACGAATGGGTATCAAGAATGG	Env (sense)	Sequencing
PA1 F5	GGAGACTATTCATCCTGGAGGAG	Env (sense)	Sequencing
PA1 R1	GCAATAGTTCCTTCTTGAGCC	Env (antisense)	Sequencing
PA1 R2	GCTCTGAGGATCTTAGGTGCAG	Env (antisense)	Sequencing
PA1 R3	CCACTAGCAGCAATAGCAGTGGC	Env (antisense)	Sequencing
PA1 R4	GGCGGTTGCACACATAGTCTAATTGG	Env (antisense)	Sequencing

ORF, Open Reading Frame
