# Supplementary material for: An EIAV field isolate reveals much higher levels of subtype variability than currently reported for the equine lentivirus family
Source: Retrovirology. 2009 Oct 20;6:95. doi: 10.1186/1742-4690-6-95 (PMC2770520; doi:10.1186/1742-4690-6-95)
Supplement: Additional File 2 — Figure S1. Genomic sequence of EIAVPA LTR population. The nucleotide sequences of the EIAVPA population and reference EIAV sequences were aligned in ClustalW to the EIAV Wyoming strain. Residues that are different from Wyoming are indicated. Transcription factor recognition sequences are boxed. Bases identical to Wyoming sequence are indicated with (white square). WYO, Wyoming; PV, EIAVPV; CHVax, Chinese vaccine stain; white square, absent base. [file 1742-4690-6-95-S2.PDF]

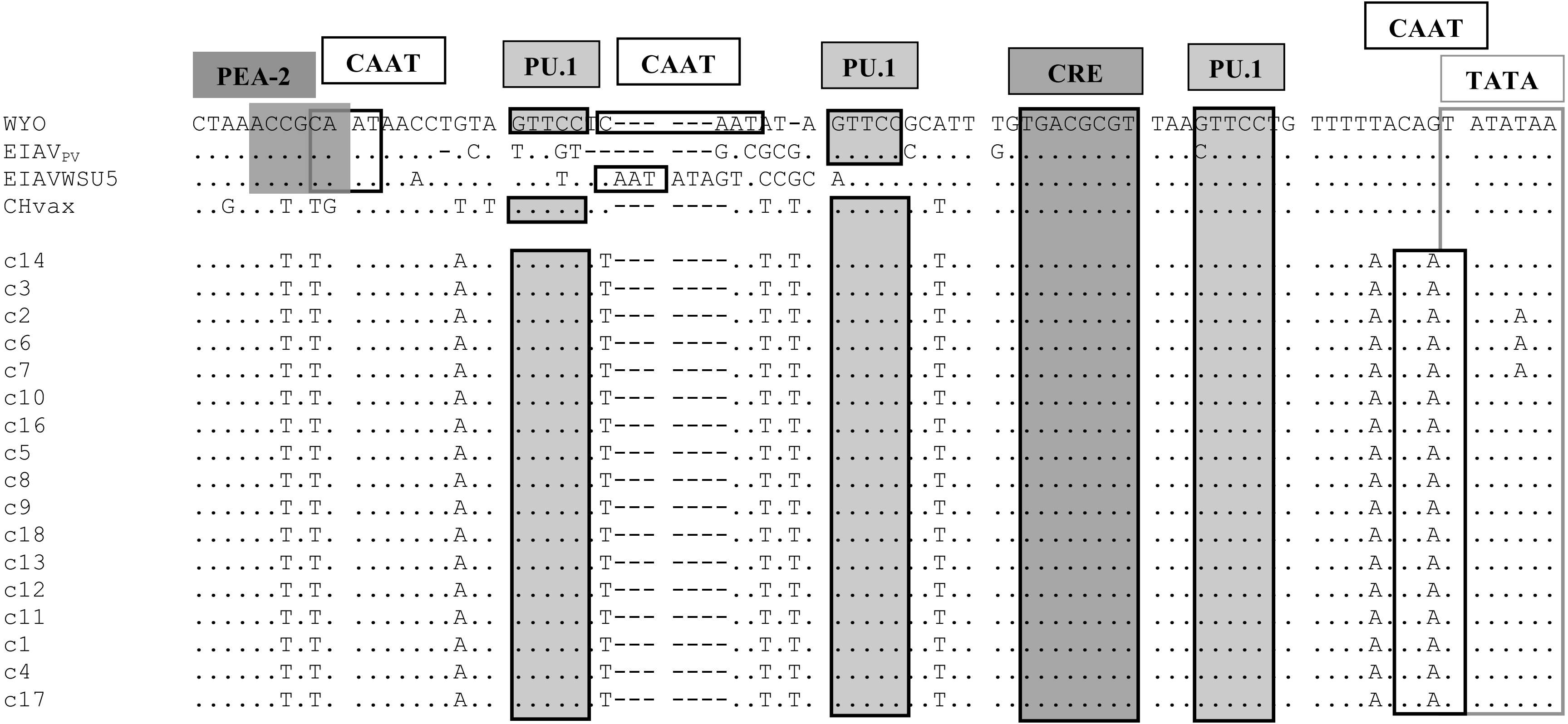
PEA-2
CAAT
PU.1
CAAT
PU.1
CRE
PU.1
CAAT
TATA
WYO CTAAACCGCA ATAACCTGTA GTTCCTC--- ---AATAT-A GTTCCGCATT TGTGACGCGT TAAGTTCCTG TTTTTACAGT ATATAA
EIAVPV ....-.C. T..GT----- ---G.CGCG. .....C.... G......... ...C...... ....
EIAVWSU5 ....A...... ...T...AAT ATAGT.CCGC A......... ....
CHvax ..G...T.TG ....T.T ....--- ----..T.T. ....T.. ....
c14 ......T.T. ....A.. ....T--- ----..T.T. ....T.. ....A..A.. ......
c3 ......T.T. ....A.. ....T--- ----..T.T. ....T.. ....A..A.. ......
c2 ......T.T. ....A.. ....T--- ----..T.T. ....T.. ....A..A.. ...A..
c6 ......T.T. ....A.. ....T--- ----..T.T. ....T.. ....A..A.. ...A..
c7 ......T.T. ....A.. ....T--- ----..T.T. ....T.. ....A..A.. ...A..
c10 ......T.T. ....A.. ....T--- ----..T.T. ....T.. ....A..A.. ......
c16 ......T.T. ....A.. ....T--- ----..T.T. ....T.. ....A..A.. ......
c5 ......T.T. ....A.. ....T--- ----..T.T. ....T.. ....A..A.. ......
c8 ......T.T. ....A.. ....T--- ----..T.T. ....T.. ....A..A.. ......
c9 ......T.T. ....A.. ....T--- ----..T.T. ....T.. ....A..A.. ......
c18 ......T.T. ....A.. ....T--- ----..T.T. ....T.. ....A..A.. ......
c13 ......T.T. ....A.. ....T--- ----..T.T. ....T.. ....A..A.. ......
c12 ......T.T. ....A.. ....T--- ----..T.T. ....T.. ....A..A.. ......
c11 ......T.T. ....A.. ....T--- ----..T.T. ....T.. ....A..A.. ......
c1 ......T.T. ....A.. ....T--- ----..T.T. ....T.. ....A..A.. ......
c4 ......T.T. ....A.. ....T--- ----..T.T. ....T.. ....A..A.. ......
c17 ......T.T. ....A.. ....T--- ----..T.T. ....T.. ....A..A.. ......
