# Supplementary material for: An EIAV field isolate reveals much higher levels of subtype variability than currently reported for the equine lentivirus family
Source: Retrovirology. 2009 Oct 20;6:95. doi: 10.1186/1742-4690-6-95 (PMC2770520; doi:10.1186/1742-4690-6-95)
Supplement: Additional File 3 — Figure S2. Genomic sequence of EIAVPA S2 population. The deduced amino acid sequences of the EIAVPA population and reference EIAV sequences were aligned in ClustalW to the EIAV Wyoming strain. Residues that are different from Wyoming are indicated by their single amino acid designations. Reported predicted nucleoporin motif, SH3 binding motif, and nuclear localization signal are underlined in the Wyoming strain. Residues identical to Wyoming sequence are indicated with (white square). Predicted N-myristilation signal is in red text and boxed. Predicted CK2 phosphorylation site is in pink text and boxed. PKC phosphorylation sites are in blue text and boxes. Predicted β-sheet is indicated with (white arrow) and boxed in orange. Predicted alpha helix is indicated with (white cylinder) and boxed in yellow. All structural predictions were performed using PredictProtein . WYO, Wyoming; PV, EIAVPV; CHVax, Chinese vaccine stain; white square, absent residue. [file 1742-4690-6-95-S3.PDF]

Nucleoporin Motif — **SH3 Domain Binding motif** — **Nuclear Localization Signal**

N-myris. site — CK2 phos site — PKC Phos sites

```
EIAVPV    MGLFGKGVTWSASHSMGGSQGESQPLLPNSQKNL--SVRRTQCFNLIVIIMTVRTAWQNRRKQETKK---
WYO       ..V..............E...................................................
EIAVWSU5  .................................P...M.K.............................
CHvax     ............L..V.V....Y...S.K.NQQTHRKEIIWYI.P...MIAIKKK..RQET.D...K..
C12       .................E......L...NT.R.TQEM.I..Y..P...LTVIKAR..KEET.D.Q.K..
C5        .................E...K..L...NT.R.TQEM.IM.Y..P...LTVIKAR..KEET.D.Q.IKT
C8        ..P..............E......L...NT.R.TQEM.IM.Y..P...LTVIKAR..KEET.D.Q.IKT
C1        .................E......L...NT.R.TQEM.IM.Y..P...LTVIKAR..KEET.D.Q.IKT
C6        .................E......L...NT.R.TQEM.IM.Y..P...LTVIKAR..KEET.D.Q.IKT
C9        .................E......L...NT.R.TQEM.IM.Y..P...LTVIKAR..KEET.D.Q.IKT
C7        .................E......L...NT.R.TQEM.IM.Y..P...LTVIKAR..KEET.D.Q.IKT
C3        .................E......L...NT.R.TQEM.IM.Y..P...LTVIKAR..KEET.D.Q.IKT
C2        .................E......L...NT.R.TQEM.IM.Y..P...LTVIKAR..KEET.D.Q.IKT
C17       .................E......L...NT.R.TQEM.IM.Y..P...LTVIKAR..KEET.D.Q.IKT
C16       .................E......L...NT.R.TQEM.IM.Y..P...LTVIKAR..KEET.D.Q.IKT
C15       .................E......L...NT.R.TQEM.IM.Y..P...LTVIKAR..KEET.D.Q.IKT
C14       .................E......L...NT.R.TQEM.IM.Y..P...LTVIKAR..KEET.D.Q.IKT
C13       .................E......L...NT.R.TQEM.IM.Y..P...LTVIKAR..KEET.D.Q.IKT
C11       .................E......L...NT.R.TQEM.IM.Y..P...LTVIKAR..KEET.D.Q.IKT
C10       .................E......L...NT.R.TQEM.IM.Y..P...LTVIKAR..KEET.D.Q.IKT
C4        .................E......L...NT.R.THEM.IM.Y..P...LTVIKAR..KEET.D.Q.IKT
C18       .................E......L...NT.R.TQEM.I..Y..P...LTVIKAR..KEET.D.Q.IKT
```
