# Supplementary material for: An EIAV field isolate reveals much higher levels of subtype variability than currently reported for the equine lentivirus family
Source: Retrovirology. 2009 Oct 20;6:95. doi: 10.1186/1742-4690-6-95 (PMC2770520; doi:10.1186/1742-4690-6-95)
Supplement: Additional File 4 — Figure S3. Genomic sequence of EIAVPA Rev second exon population. The deduced amino acid sequences of the EIAVPA population and reference EIAV sequences were aligned in ClustalW to the EIAV Wyoming strain. Residues that are different from Wyoming are indicated by their single amino acid designations. Reported activation domain, RNA binding site and nuclear exportation signal are underlined in the Wyoming sequence and are boxed in the EIAVPA population and reference EIAV sequences. Residues identical to Wyoming sequence are indicated with (white square). Glycosylation sites are colored orange. WYO, Wyoming; PV, EIAVPV; CHVax, Chinese vaccine stain; white square, absent residue. [file 1742-4690-6-95-S4.PDF]

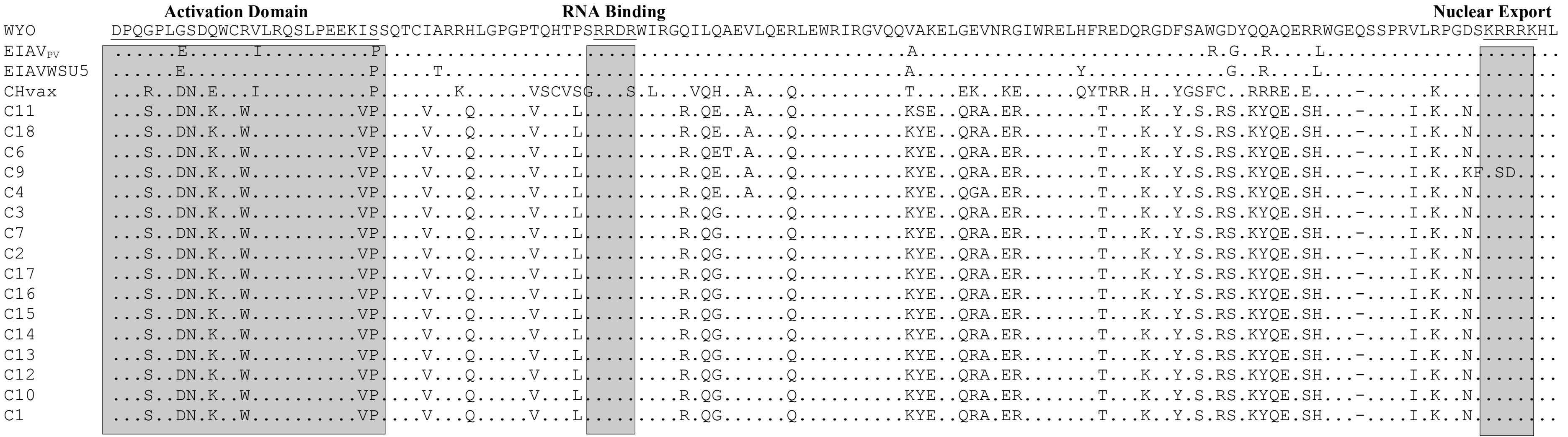

Activation Domain
RNA Binding
Nuclear Export
WYO DPQGPLGSDQWCRVLRQSLPEEKISSQTCIARRHLGPGPTQHTPSRRDRWIRGQILQAEVLQERLEWRIRGVQQVAKELGEVNRGIWRELHFREDQRGDFSAWGDYQQAQERRWGEQSSPRVLRPGDSKRRRKHL
EIAVPV
EIAVWSU5
CHvax
C11
C18
C6
C9
C4
C3
C7
C2
C17
C16
C15
C14
C13
C12
C10
C1
