# Supplementary material for: An EIAV field isolate reveals much higher levels of subtype variability than currently reported for the equine lentivirus family
Source: Retrovirology. 2009 Oct 20;6:95. doi: 10.1186/1742-4690-6-95 (PMC2770520; doi:10.1186/1742-4690-6-95)
Supplement: Additional File 5 — Figure S4. Genomic sequence of EIAVPA Env gp45 population. The deduced amino acid sequences of the EIAVPA population and reference EIAV sequences were aligned in ClustalW to the EIAV Wyoming strain. Residues that are different from Wyoming are indicated by their single amino acid designations. The transmembrane domain is boxed. Residues identical to Wyoming sequence are indicated with (white square). Glycosylation sites are colored orange. WYO, Wyoming; PV, EIAVPV; CHVax, Chinese vaccine stain; white square, absent residue. [file 1742-4690-6-95-S5.PDF]

```
WYO     DFGVSAVVAAIVAATAIAASATMSYVALTEVNKIMEVQNHTFEVENSTLNGMDLIEQQIKILYAMILQTHADVQLLKERQQVEETFNLIGCIERTHIFCHTGHPWNMSWGHLNESTQWDDWVSKMEDLNQDILTTLHVARNNLAQSMITF
PV      ...I..I.................................................R.......................................V.................................E...I..G............
WSU5    ...I..I.................................................R.......................................V.................................E......G............
CHVax   ...I..II.................I.......LDS..........N.I.SIE.T.E..H.....V............Q.KI............S.T.........E...Q..D........D...N..H.......T.....E......
c11     ...I..II............G..A.I....A..LAD..........N.....EI..K..H.....V........I...K.HI...........Q..T.........D...Q..DT....E...R..SY.................A....
c5      ...I..II............G..A.I....A..LAD..........N..S..EI..K..H.....V........I...K.HI...........Q..T.........D...Q..DT....E...R..SY.................A....
c8      ...I..II............G..A.I....A..LAD..........N.....EI..K..H.....V........I...K.HI...........Q..T.........D...Q..DT....E...R..SY.................A....
c18     ...I..II............G..A.I....A..LAD..........N..S..EI..K..H.....V........I...K.HI...........Q..T.........D...Q..DT....E...R..SY.................A....
c6      ...I..II............G..A.I....A..LAD..........N.....EI..K..H.....V........I...K.HI...........Q..T.........D...Q..DT....E...R..SY.................A....
c4      ...I..II............G..A.I....A..LAD..........N.....EI..K..H.....V........I...K.HI...........Q..T.........D...Q..DT....E...R..SY.................A....
c9      ...I..II..........V.G..A.I....A..LAD..........N..S..EI..K..H.....V........I...K.HI...........Q..T.........D...Q..DT....E...R..SY.................A....
c3      ...I..II............G..A.I....A..LAD..........N.....EI..K..H.....V........I...K.HI...........Q..T.........D...Q..DT....E...R..SY.................A....
c2      ...I..II............G..A.I....A..LAD..........N.....EI..K..H.....V........I...K.HI...........Q..T.........D...Q..DT....E...R..SY.................A....
c7      ...I..II............G..A.I....A..LAD..........N.....EI..K..H.....V........I...K.HI...........Q..T.........D...Q..DT....E...R..SY.................A....
c16     ...I..II............G..A.I....A..LAD..........N.....EI..K..H.....V........I...K.HI...........Q..T.........D...Q..DT....E...R..SY.................A....
c14     ...I..II............G..A.I....A..LAD..........N.....EI..K..H.....V........I...K.HI...........Q..T.........D...Q..DT....E...R..SY.................A....
c17     ...I..II............G..A.I....A..LAD..........N.....EI..K..H.....V........I...K.HI...........Q..T.........D...Q..DT....E...R..SY.................A....
c15     ...I..II............G..A.I....A..LAD..........N.....EI..K..H.....V........I...K.HI...........Q..T.........D...Q..DT....E...R..SY.................A....
c13     ...I..II............G..A.I....A..LAD..........N.....EI..K..H.....V........I...K.HI...........Q..T.........D...Q..DT....E...R..SY.................A....
c1      ...I..II............G..A.I....A..LAD..........N.....EI..K..H.....V........I...K.HI...........Q..T.........D...Q..DT....E...R..SY.................A....
c10     ...I..II............G..A.I....A..LAD..........N.....EI..K..H.....V........I...K.HI...........Q..T.........D...Q..DT....E...R..SY.....I...........A....
c12     ...I..II............G..A.I....A..LAD..........N..S..EI..K..H.....V........I...K.HI...........Q..T.........D...Q..DT....E...R..SY.................A....
```

Transmembrane Domain

```
WYO     NTPDSIAQFGKDLWSHIANWIPGLGASIIKYIVMFLLIYLLLTSSPKILRALWEVTSGAGSSGSRYLKKKFHRKHASREDTWDQAQHNIHLAGVTGGSGDKYYRQKYSRNDWNGESEEYNRWPKSWVKSIEAFGESYISEKTKGEISQPG
PV      .................G...................................K..................H..............................K..C..............R............................
WSU5    .................G...................................K..................H..............................K.................R............................
CHVax   ...........NI...................MLL..V.M....A.....G.LTTM......A....R.RY.HR....GNI.A.V.YHAY..DE.H.....SNMR.L...N..........RQ.N.K.L.KRS..N.NTHEDNMGTMGRF
c11     .....V.....NI.D..T..........T...............A.......LTTI......A.HF...RY..R.VWQ..NL..G.YS....D..A.LE.E.K...HF.SN......GS.KNLR.SKGLMREY.G..T.LR..K.TIH..
c5      .....V.....NI.D.............T.........IW....A.......LTTI......A.HF...RY..R.VWQ..NL..G.YS....D..A.LE.E.K...HF.SN......GS.KNLR.SKGLMREY.G..T.LR..K.TIH..
c8      .....V.....NI.D.............T...............A.......LTTI......A.HF...RY..R.VWQ..NL..G.YS....D..A.LE.E.K...HF.SN......GS.KNLR.SKGLMREY.G..T.LR..K.TIH..
c18     .....V.....NI.D.............T...............A.......LTTI......A.HF...RY..R.VWQ..NL..G.YS....D..A.LE.E.K...HF.SN......GS.KNMR.SKGLMREY.G..T.LR..K.TIH..
c6      .....V.....NI.D.............T...............A.......LTTI......A.HF...RY..R.VWQ..NL..G.YS....D..A.LE.E.K...HF.SN......GS.KNMR.SKGLMREY.G..T.LR..K.TIH..
c4      .....V.....NI.D.............T..........V....A.......LTTI......A.HF...RY..R.VWQ..NL..G.YS....D..A.LE.E.K...HF.SN......GS.KNMR.SKGLMREY.G..T.LR..K.TIH..
c9      .....V.....NI.D..........V..T...............A.......LTTI......A.HF...RY..R.VWQ..NL..G.YS....D..A.LE.E.K...HF.SN......GS.KNMR.SKGLMREY.G..T.LR..K.TIH..
c3      .....V.....NI.D.............T...............A.......LTTI......A.HF...RY..R.VWQ..NL..G.YS....D..A.LE.E.KGR..F.SN......GS.KNMR.SKGLMREY.G..T.LR..K.TIH..
c2      .....V.....NI.D.............T...............A.......LTTI......A.HF...RY..R.VWQ..NL..G.YS....D..A.LE.E.KGR..F.SN......GS.KNMR.SKGLMREY.G..T.LR..K.TIH..
c7      .....V.....NI.D.............T...............A.......LTTI......A.HF...RY..R.VWQ..NL..G.YS....D..A.LE.E.KGR..F.SN......GS.KNMR.SKGLMREY.G..T.LR..K.TIH..
c16     .....V.....NI.D.............T...............A.......LTTI......A.HF...RY..R.VWQ..NL..G.YS....D..A.LE.E.KGR..F.SN......GS.KNMR.SKGLMREY.G..T.LR..K.TIH..
c14     .....V.....NI.D.............T...............A.......LTTI......A.HF...RY..R.VWQ..NL..G.YS....D..A.LE.E.KGR..F.SN......GS.KNMR.SKGLMREY.G..T.LR..K.TIH..
c17     .....V.....NI.D.............T...............A.......LTTI......A.HF...RY..R.VWQ..NL..G.YS....D..A.LE.E.KGR..F.SN......GS.KNMR.SKGLMREY.G..T.LR..K.TIH..
c15     .....V.....NI.D.............T...............A.......LTTI......A.HF...RY..R.VWQ..NL..G.YS....D..A.LE.E.KGR..F.SN......GS.KNMR.SKGLMREY.G..T.LR..K.TIH..
c13     .....V.....NI.D.............T...............A.......LTTI......A.HF...RY..R.VWQ..NL..G.YS....D..A.LE.E.KGR..F.SN......GS.KNMR.SKGLMREY.G..T.LR..K.TIH..
c1      .....V.....NI.D.............T...............A.......LTTI......A.HF...RY..R.VWQ..NL..G.YS....D..A.LE.E.KGR..F.SN......GS.KNMR.SKGLMREY.G..T.LR..K.TIH..
c10     .....V.....NI.D.............T...............A.......LTTI......A.HF...RY..R.VWQ..NL..G.YS....D..A.LE.E.KGR..F.SN......GS.KNMR.SKGLMREY.G..T.LR..K.TIH..
c12     .....V.....NI.D.............T...............A.......LTTI......A.HF...RY..R.VWQ..NL..G.YS....D..A.LE.E.KGR..F.SN......GS.KNMR.SKGLMREY.G..T.LR..K.TIH..
```

```
WYO     ATINKHKNGAGGNNPHQGSLDL-EIRSEGGNIYDCCIKAQEGTLAIPCCGFPLWLFWGLLIILGRIAGYGLRGLAVIIRICIRGLNLIFEIIRKMLDYIGRALNPGTSHVSMPQY-V
PV      .A..E....S............-....................................V..V....................................................-.
WSU5    .A..E....S............-....................................V..V..................T.................................-.
CHVax   V.TAAE.KNV.V.-......N.-..Q.............................L...I.....LL......I.K..M.LAK...V.ITRL..LC....KM...A....T....D.
c11     GAT.TK.KVT.E.-......N.-..QND...............I.......L.............LI..V.....K.LMNVGK..SMLL.V..RV.....K....ASQ.......-.
c5      GAT.TK.KVT.E.-......N.-..QND...............I.......L.............LI..V.....K.LMNVGK..SMLL.V..RV.....K....ASQ...T...-.
c8      GAT.TK.KVT.E.-......N.-..QND...............I.......L.............LI..V.....K.LMNVGK..SMLL.V..RV.....K....ASQ.......-.
c18     GAT.TK.KVT.E.-......N.-..QND...............I.......L.............LI..V.....K.LMNVGK..SMLL.V..RV.....K....ASQ.......-.
c6      GAT.TK.KVT.E.-......N.-..QND...............I.......L.............LI..V.....K.LMNVGK..SMLL.V..RV...L.K....ASQ.......-.
c4      GAT.TK.KVT.E.-......N.-..QND...............I.......L.............LM..V.....K.LMNVGK..SMLL.V..RV.....K....ASQ.......-.
c9      GAT.TK.KVT.E.-......N.GNSKAT.......G.......I.......L.............LI..V.....K.LMNVGK..SMLL.V..RV.....K....ASQ.......-.
c3      GVT.TK.KVT.E.-......N.-..QND...............I.......L.............LI..V.....K.LMNVGK..SMLL.V..RV...F.K....ASQ.......-.
c2      GVT.TK.KVT.E.-......N.-..QND...............I.......L.............LI..V.....K.LMNVGK..SMLL.V..RV...L.K....ASQ.......-.
c7      GVT.TK.KVT.E.-......N.-..QND...............I.......L.............LI..V.....K.LMNVGK...MLL.V..RV...L.K....ASQ.......-.
c16     GVT.TK.KVT.E.-......N.-..QND...............I.......L.............LI..V.....K.LMNVGK..SMLL.V..RV.....K....ASQ.......-.
c14     GVT.TK.KVT.E.-......N.-..QND...............I.......L.............LI..V.....K.LMNVGK..SMLL.V..RV.....K....ASQ.......-.
c17     GVT.TK.KVT.E.-......N.-..QND...............I.......L.............LI..V.....K.LMNVGK..SMLL.V..RV.....K....ASQ.......-.
c15     GVT.TK.KVT.E.-......N.-..QND...............I.......L.............LI..V.....K.LMNVGK..SMLL.V..RV.....K....ASQ.......-.
c13     GVT.TK.KVT.E.-......N.-..QND...............I.......L.............LI..V.....K.LMNVGK..SMLL.V..RV.....K....ASQ.......-.
c1      GVT.TK.KVT.E.-......N.-..QND...............I.......L.............LI..V.....K.LMNVGK..SMLL.V..RV.....K....ASQ.......-.
c10     GVT.TK.KVT.E.-......N.-..QND...............I.......L.............LI..V.....K.LMNVGK..SMLL.V..RV.....K....ASQ.......-.
c12     GVT.TK.KVT.E.-......N.-..QND...............I.......L.............LI..V.....K.LMNVGK..SMLL.V..RV.....K....ASQ.......-.
```
